# A Novel Approach to the Treatment of Pembrolizumab-induced Psoriasis Exacerbation: A Case Report

**DOI:** 10.7759/cureus.5824

**Published:** 2019-10-02

**Authors:** Elio P Monsour, Joshua Pothen, Rama Balaraman

**Affiliations:** 1 Internal Medicine, University of Central Florida / Ocala Regional Medical Center, Ocala, USA; 2 Internal Medicine, General Medical Education, Ocala Regional Medical Center, Ocala, USA; 3 Hematology and Oncology, Florida Cancer Affiliates / Ocala Oncology, Ocala, USA

**Keywords:** immunotherapy in lung cancer, psoriasis, pembrolizumab, oncology, dermatology, pd-l1 inhibitor

## Abstract

While immune checkpoint inhibitors have been groundbreaking for cancer treatment, there are many reported cases of patients undergoing immunotherapy who have discontinued or temporarily interrupted treatment due to the development of autoimmune-related adverse effects. Here, we present a 63-year-old female with a history of psoriasis (in spontaneous remission) and newly diagnosed poorly differentiated lung adenocarcinoma (pTXN3M1a) who experienced a severe flare-up of her psoriasis three months after initiating single-agent pembrolizumab. The patient was initially treated with topical clobetasol propionate ointment, however, due to minimal response to this regimen, the patient was commenced on secukinumab; an IL-17 inhibitor. To our knowledge, this is the first case of the successful use of secukinumab for the treatment of immunotherapy-induced psoriasis. More importantly, immunotherapy with pembrolizumab was continued successfully with the co-administration of secukinumab without complications or the recurrence of non-small cell lung cancer (NSCLC).

## Introduction

Immune checkpoint inhibitors have been a breakthrough for cancer therapy in recent years, especially for the treatment of metastatic non-small cell lung cancer (NSCLC). Pembrolizumab is immunotherapy that works by blocking the interaction between the programmed death-ligand 1 (PD-L1) receptor on T-cells with the PD-L1 and PD-L2 ligands on tumor cells [1]. Consequently, this promotes T-cell reactivation and restored immune response, rather than T-cell deactivation. However, immunotherapy is associated with increased immune-related adverse effects. Early discontinuation of immunotherapy may pose a risk to treatment outcomes, and, thus, recognition and management of its underlying side effects are crucial. We report an unusual case of pembrolizumab-induced psoriasis exacerbation treated effectively with secukinumab.

## Case presentation

A 63-year-old female patient with a history of hypothyroidism, gastroesophageal reflux disease (GERD), hypertension, and psoriasis initially presented to the clinic with complaints of right hip pain. Magnetic resonance imaging (MRI) of the right hip revealed edema in the trochanteric bursa and a mass measuring 3.6 x 1.8 cm in size. Two adjacent nodules in the pelvis concerning for adenopathy were also discovered. Subsequent whole-body positron emission tomography-computed tomography (PET/CT) scan demonstrated hypermetabolic adenopathy in the neck, chest, and pelvis. Lymph node biopsy of the pelvis established the histological diagnosis of poorly differentiated lung adenocarcinoma, clinically staged as TXN3M1a NSCLC (stage IVb). Genomic molecular analysis showed programmed death-ligand 1 (PD-L1) positivity with >50% expression without known estimated glomerular filtration rate (EGFR) or ALK genomic tumor aberrations.

**Figure 1:**
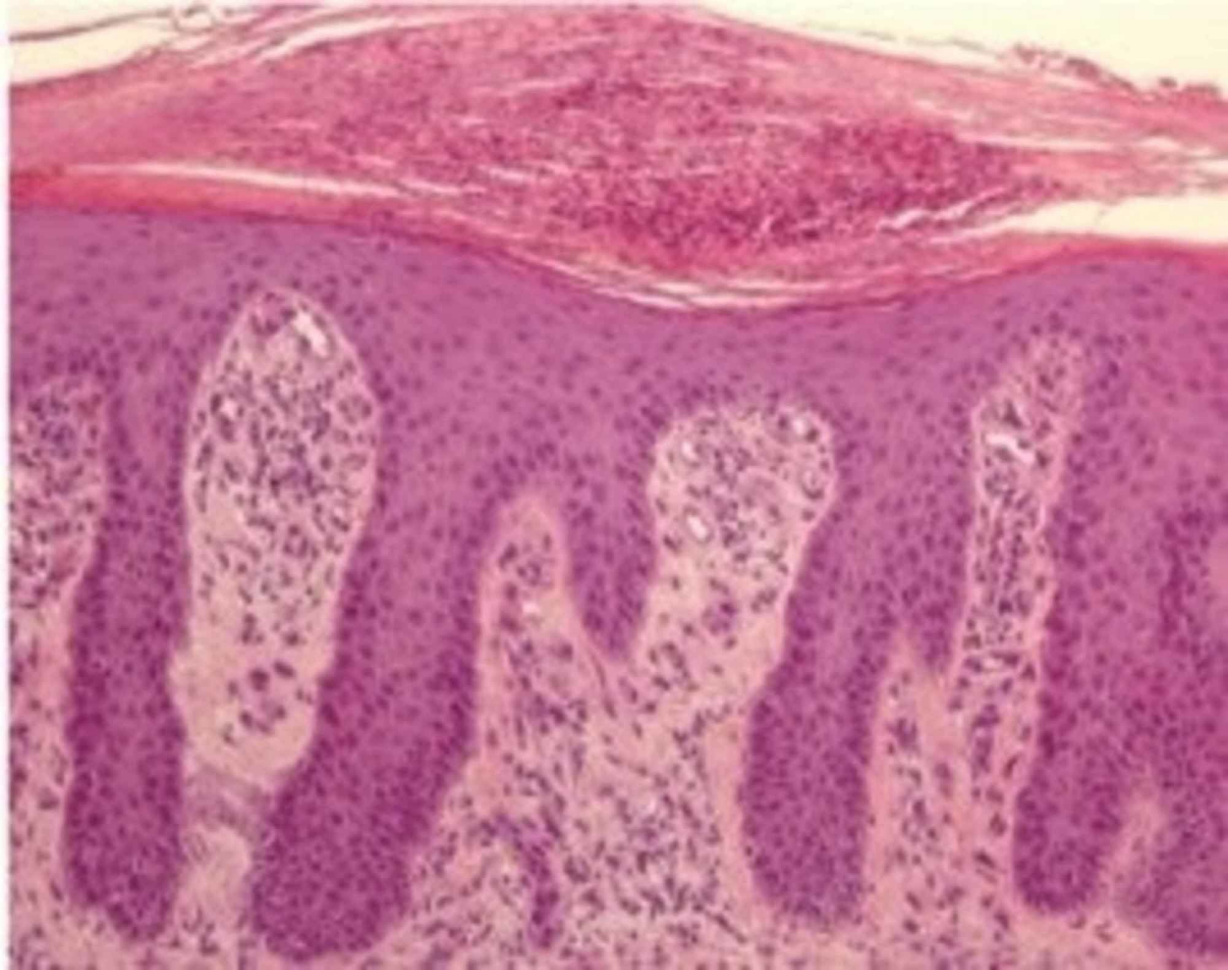
Histopathology of skin lesions using hematoxylin and eosin staining (approximate magnification 200x) consistent with psoriasis Adapted from Galluzo et al. [2].

**Figure 2:**
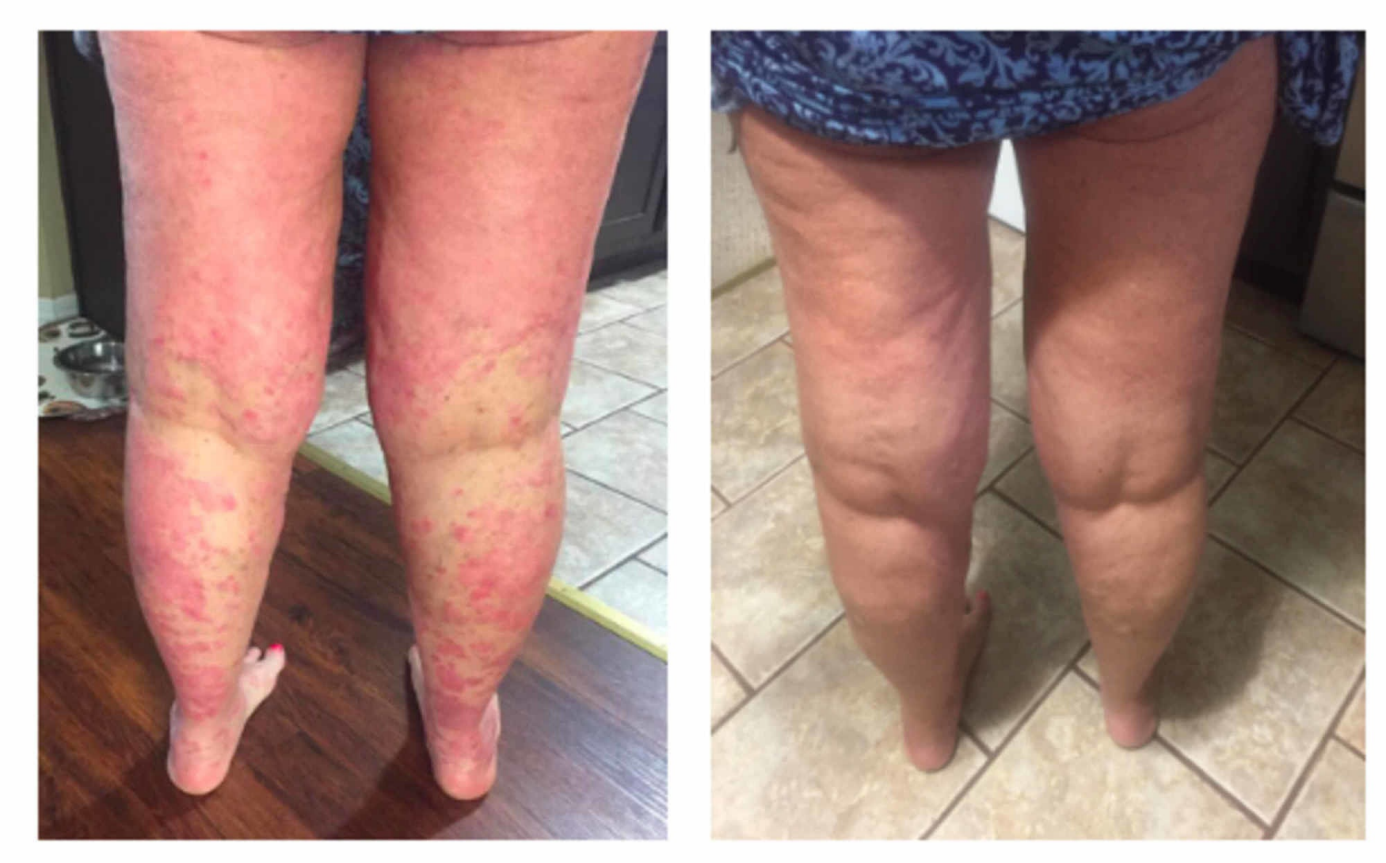
Before and after photos following treatment with secukinumab

Prior to the initiation of immunotherapy with pembrolizumab 200 mg intravenous (IV) every three weeks, the patient had no active psoriatic lesions and had been in spontaneous remission of her psoriasis for the past couple of years. However, three months following immunotherapy, the patient developed diffuse dermatitis on her left ventral proximal forearm, anterior proximal thigh, arms, legs, and trunk. The patient was referred to dermatology, and a shave biopsy was performed. Pathology showed severe plaque psoriasis, most probably induced by pembrolizumab (Figure 1). She was started on 0.05% clobetasol propionate cream but failed to respond to treatment after four weeks. Computed tomography (CT) of the chest, abdomen, and pelvis showed no findings of recurrence or metastatic disease in the chest. Following a negative hepatitis panel and interferon-gamma release assay, the patient was commenced on secukinumab and, gradually, the patient’s psoriasis was ameliorated. Given the clinical response to secukinumab for psoriasis, pembrolizumab was continued without further complications (Figure 2).

## Discussion

This case illustrates plaque psoriasis exacerbation following treatment with pembrolizumab, successfully treated with secukinumab. Other etiologies of psoriasis flare-up were ruled out, including but not limited to infections, alcohol intake, skin injury, or use of other medications associated with worsening of psoriasis. The patient’s quality of life was severely affected due to her extensive psoriatic lesions, even though she had no evidence of metastatic disease or recurrence of NSCLC. The etiology of psoriasis and other autoimmune adverse events secondary to immunotherapy is said to be due to T-cell activation-enhanced autoimmunity [3].

**Figure 3:**
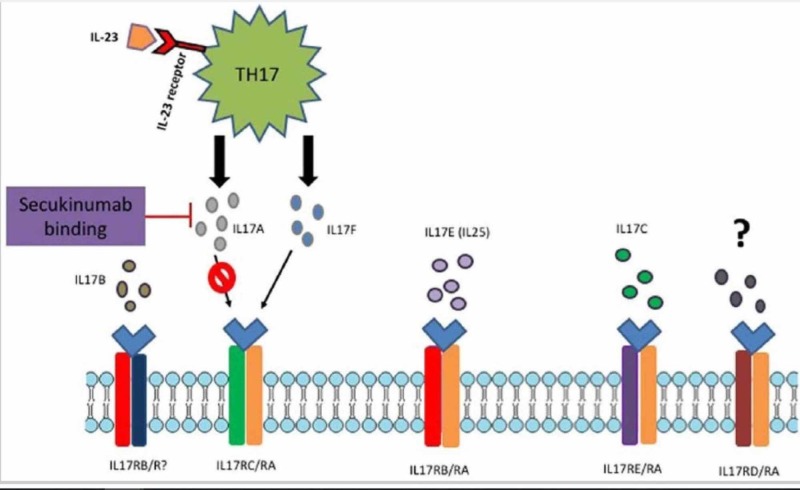
Secukinumab mechanism of action Adapted from Frieder et al. [4].

Often, treatment of these autoimmune conditions requires the use of immunosuppression therapy and interruption or complete discontinuation of immunotherapy, which may all pose cancer treatment risk outcomes. Secukinumab works by selectively targeting IL-17A and blocks its interaction with the IL-17 receptor, thus inhibiting downstream pro-inflammatory cytokine production (Figure 3) [4].^ ^A review of published literature via PubMed until July 2019 has demonstrated approximately 34 cases of psoriasis due to anti-PD-1/PD-L1 therapy [5]; however, to our knowledge, this is the first case of the successful use of secukinumab for the treatment of immunotherapy-induced psoriasis. More importantly, immunotherapy with pembrolizumab was continued successfully with the co-administration of secukinumab without complications or recurrence of NSCLC.

## Conclusions

We report an interesting case of a patient with PD-L1 positive advanced non-small cell lung cancer who developed severe exacerbation of her psoriasis secondary to treatment with pembrolizumab, which achieved sustained response to secukinumab. Clinicians should be cognizant of autoimmune-related adverse effects following immunotherapy and promptly recognize these complications. Optimal treatment and duration are in question, and further research should be conducted to assess long-term effects.
